# Case report: multiple and atypical amoebic cerebral abscesses resistant to treatment

**DOI:** 10.1186/s12879-020-05391-y

**Published:** 2020-09-14

**Authors:** Joaquin Alvaro Victoria-Hernández, Anayansi Ventura-Saucedo, Aurelio López-Morones, Sandra Luz Martínez-Hernández, Marina Nayeli Medina-Rosales, Martín Muñoz-Ortega, Manuel Enrique Ávila-Blanco, Daniel Cervantes-García, Luis Fernando Barba-Gallardo, Javier Ventura-Juárez

**Affiliations:** 1 Departamento de Anatomía Patológica, Hospital General de Zona 3 IMSS Jesús María, Prolongación General Ignacio Zaragoza 905, Jesús María, CP 20908 Aguascalientes, AGS Mexico; 2 Departamento de Anestesiología, Hospital General de Zona 3 IMSS Jesús María, Prolongación General Ignacio Zaragoza 905, Jesús María, CP 20908 Aguascalientes, AGS Mexico; 3 Departamento de Neurocirugía, Hospital General de Zona 3 IMSS Jesús María, Prolongación General Ignacio Zaragoza 905, Jesús María, CP 20908 Aguascalientes, AGS Mexico; 4 Departamento de Morfología, Centro de Ciencias Básicas, Universidad Autónoma de Aguascalientes, Ed. 202 Av Universidad # 940, Ciudad Universitaria, CP 20131 Aguascalientes, AGS Mexico; 5 Departamento de Química, Centro de Ciencias Básicas, Universidad Autónoma de Aguascalientes, CP 20131 Aguascalientes, AGS Mexico; 6 Departamento de Microbiología, Centro de Ciencias Básicas, Universidad Autónoma de Aguascalientes, CP 20131 Aguascalientes, AGS Mexico; 7 Consejo Nacional de Ciencia y Tecnología, CONACYT, 03940 Ciudad de México, Mexico; 8 Departamento de Optometría, Centro de Ciencias de la Salud, Universidad Autónoma de Aguascalientes, CP 20131 Aguascalientes, AGS Mexico

**Keywords:** Cerebral amoebiasis, *Entamoeba histolytica*, NMRI, PCR, 140 kDa fibronectin receptor, Brain abscess

## Abstract

**Background:**

The parasite *Entamoeba histolytica* is the causal agent of amoebiasis, a worldwide emerging disease. Amebic brain abscess is a form of invasive amebiasis that is both rare and frequently lethal. This condition always begins with the infection of the colon by *E. histolytica* trophozoites, which subsequently travel through the bloodstream to extraintestinal tissues.

**Case presentation:**

We report a case of a 71-year-old female who reported an altered state of consciousness, disorientation, sleepiness and memory loss. She had no history of hepatic or intestinal amoebiasis. A preliminary diagnosis of colloidal vesicular phase neurocysticercosis was made based on nuclear magnetic resonance imaging (NMRI). A postsurgery immunofluorescence study was positive for the 140 kDa fibronectin receptor of *E. histolytica*, although a serum analysis by ELISA was negative for IgG antibodies against this parasite. A specific *E. histolytica* 128 bp rRNA gene was identified by PCR in biopsy tissue. The final diagnosis was cerebral amoebiasis. The patient underwent neurosurgery to eliminate amoebic abscesses and was then given a regimen of metronidazole, ceftriaxone and dexamethasone for 4 weeks after the neurosurgery. However, a rapid decline in her condition led to death.

**Conclusions:**

The present case of an individual with a rare form of cerebral amoebiasis highlights the importance of performing immunofluorescence, NMRI and PCR if a patient has brain abscess and a poorly defined diagnosis. Moreover, the administration of corticosteroids to such patients can often lead to a rapid decline in their condition.

## Background

*Entamoeba histolytica* is the causal agent of amoebiasis*,* an emerging disease found worldwide [1]. This disease is prevented by improving sanitation. Although usually manifested in the human intestine [2], this agent can spread to the liver or brain and generate abscesses. Over 25 years ago, the molecular characterization of *E. histolytica* [3] provided an epidemiological pyramid in which 10% of the world population is infected by a noninvasive form of the parasite and 1% by an invasive form [2]. According to epidemiological evidence from PCR, amoebiasis ranks among the 20 most common causes of death in Mexico [1].

Cerebral amoebiasis, a very rare form of the disease, is often difficult to diagnose due to the limited availability of proper diagnostic tools. This disease has rarely been reported (see the following reviews: [4–6]), with only 133 documented cases [7–10], which chiefly occurred in young and middle-aged adults (22–65 years old) suffering from hepatic abscess or intestinal amoebiasis. The associated symptoms are headaches, altered mental status, meningeal disorders, seizure and vomiting. Of the 133 cases in the literature, 10 were given a timely diagnosis and adequate treatment (metronidazole and dehydroemetine), resulting in complete recovery [7]. In the other cases, it was not possible to make an accurate diagnosis. Consequently, proper therapy was not provided, and the patients died [10]. In Japan, a country with excellent public health measures, persons found to have cerebral amoebiasis are offered highly efficacious treatments that often lead to total recovery [7, 8]. We herein present a case report of cerebral abscesses caused by *E. histolytica* in a woman with no history of hepatic or intestinal amoebiasis. Her clinical condition very rapidly declined and ended in death.

## Case presentation

**Fig. 1:**
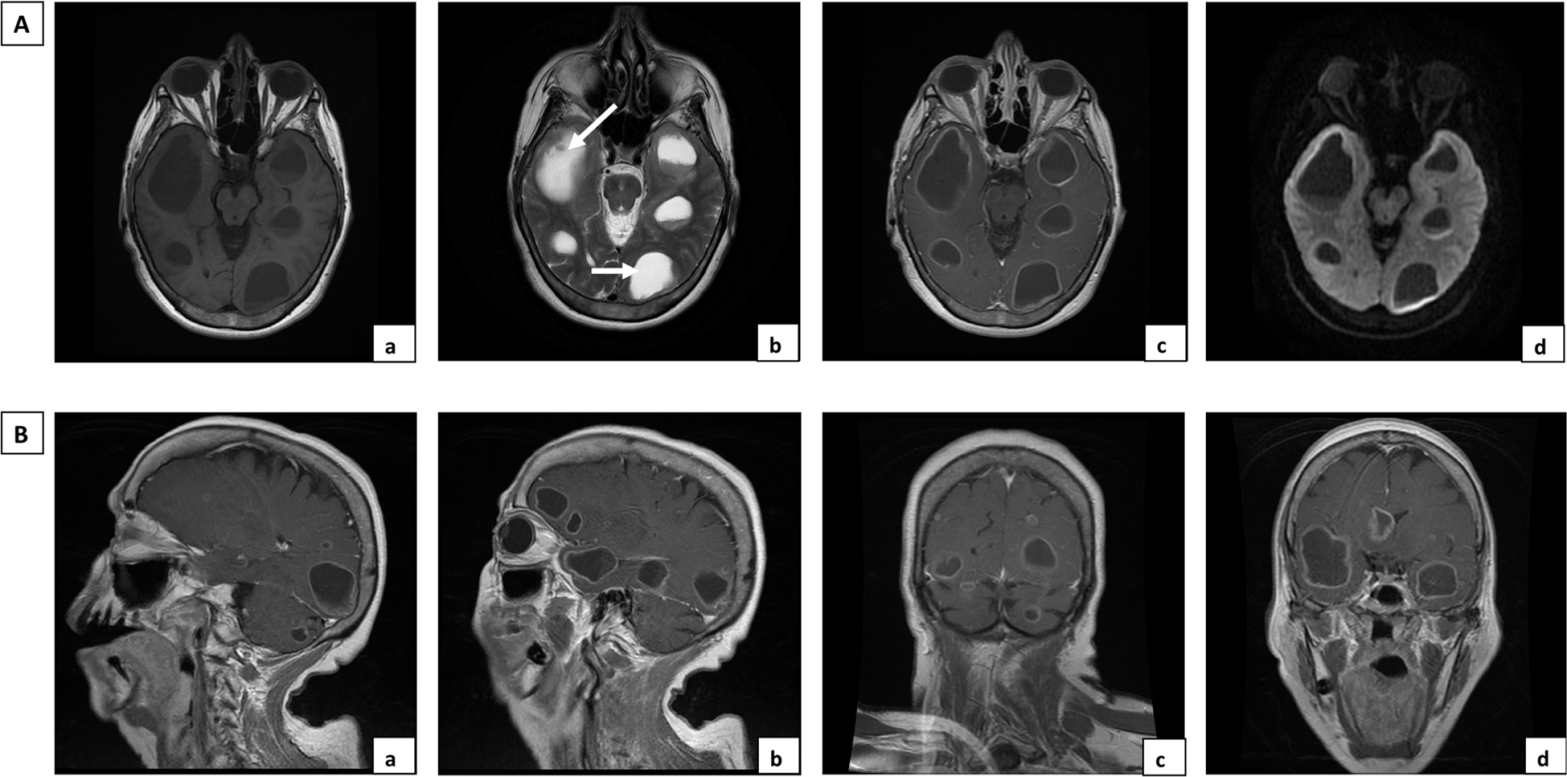
Cranial nuclear magnetic resonance image. A. Multiple cystic lesions with ring enhancement after contrast administration, without restricted diffusion, in temporal and occipital lobes. (a) Axial T1SE, (b) Axial T2 Propeller, (c) Axial T1 SE + gadolinium, (d) Axial DWI. B. Gadolinium contrast brain NMRI showing ring-enhanced lesions with multilobar distribution. (a) and (b) Sagittal T1 SE + gadolinium, (c) coronal T1 SE + gadolinium with supra- and infratentorial lesions, (d) coronal T1 SE + gadolinium with temporal and intraventricular lesions

The 71-year-old female was a healthy housewife with no record of medical interventions. She had a family history of cerebral cancer. August 4, 2018, marked the onset of a series of symptoms, including an altered state of consciousness, disorientation and sleepiness and no presence of fever. She first consulted a doctor in private practice and was diagnosed with transient cerebral ischemia. The onset of memory loss and the persistence of the previous symptoms led the patient to seek medical attention in a public hospital where she was admitted and blood analysis was performed. The only alteration in the basic blood panel was high blood pressure, with a value of 149/100 mmHg. Pallor was observed in the skin and integuments. Neurological examination only showed cognitive impairment with bradypsychia, disorientation in time and space and difficulty in carrying out simple calculations, with no fever or meningeal signs. Nuclear magnetic resonance imaging using gadolinium contrast (NMRI) of the brain revealed multiple bilateral cystic lesions containing varying amounts of fluid (white arrows in Fig. 1Ab). The lesions were detected in several brain locations: the frontal, temporal and occipital lobes (Fig. 1Aa-d) and in the supra- and infratentorial zones (Fig. 1Ba-d). Since some of the lesions were compatible with a diagnosis of colloidal vesicular phase neurocysticercosis, because the hospital did not have a stereotaxic frame and due to the multiple locations of the abscesses, the patient was submitted to a right temporal craniotomy under general anesthesia on August 25, 2018. The layers of tissue were separated, working from the skin to the brain and through the superior temporal sulcus. A cyst (without capsule) was removed from the right temporal lobe, which had a diameter of approximately 5 mm, contents with a milky not suppurative aspect and a periphery composed of soft whitish tissue (see supplementary video). A fragment of biopsy-extracted tissue was fixed in formaldehyde at 10% to be processed for histopathological examination. The surgical lesion was closed in layers from the dura to the skin.

The patient was discharged on September 3, 2018 with a diagnosis of probable neurocysticercosis and possible hydatid cysts. The sample was not grown in bacterial culture, and the medical ethics committee decided to perform a histopathological study and ELISA to obtain a definitive diagnosis.

**Fig. 2:**
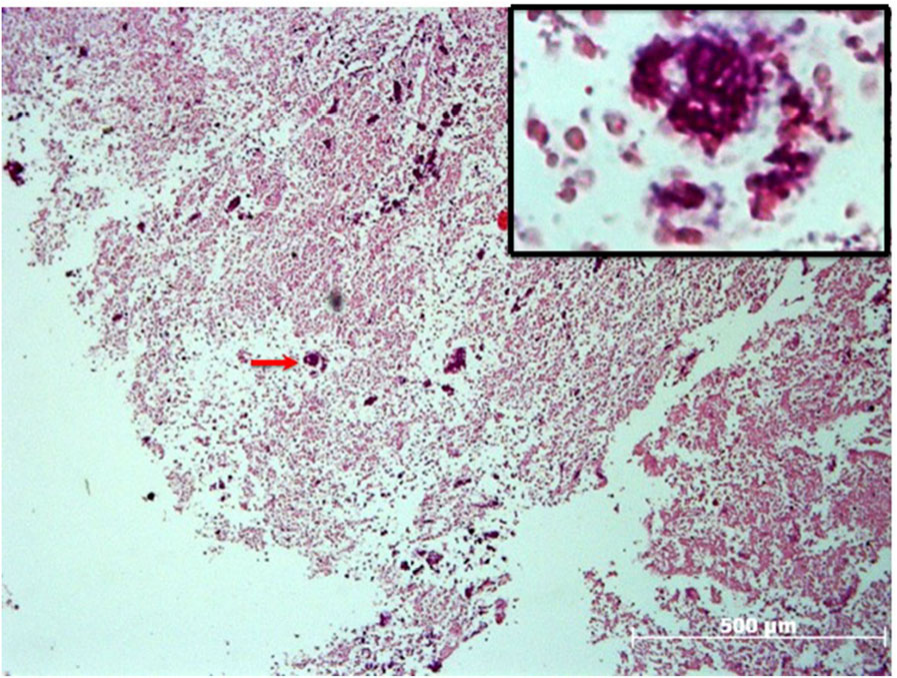
*E. histolytica* trophozoites are revealed in amoebic brain abscesses by histopathological study. A broad area of necrotic nerve tissue can be observed. Trophozoites are widely distributed in the abscess (red arrow), as illustrated by the light microscopic images (X50). An amoebic trophozoite is shown with H&E staining (box in the upper right corner, X400)

**Fig. 3:**
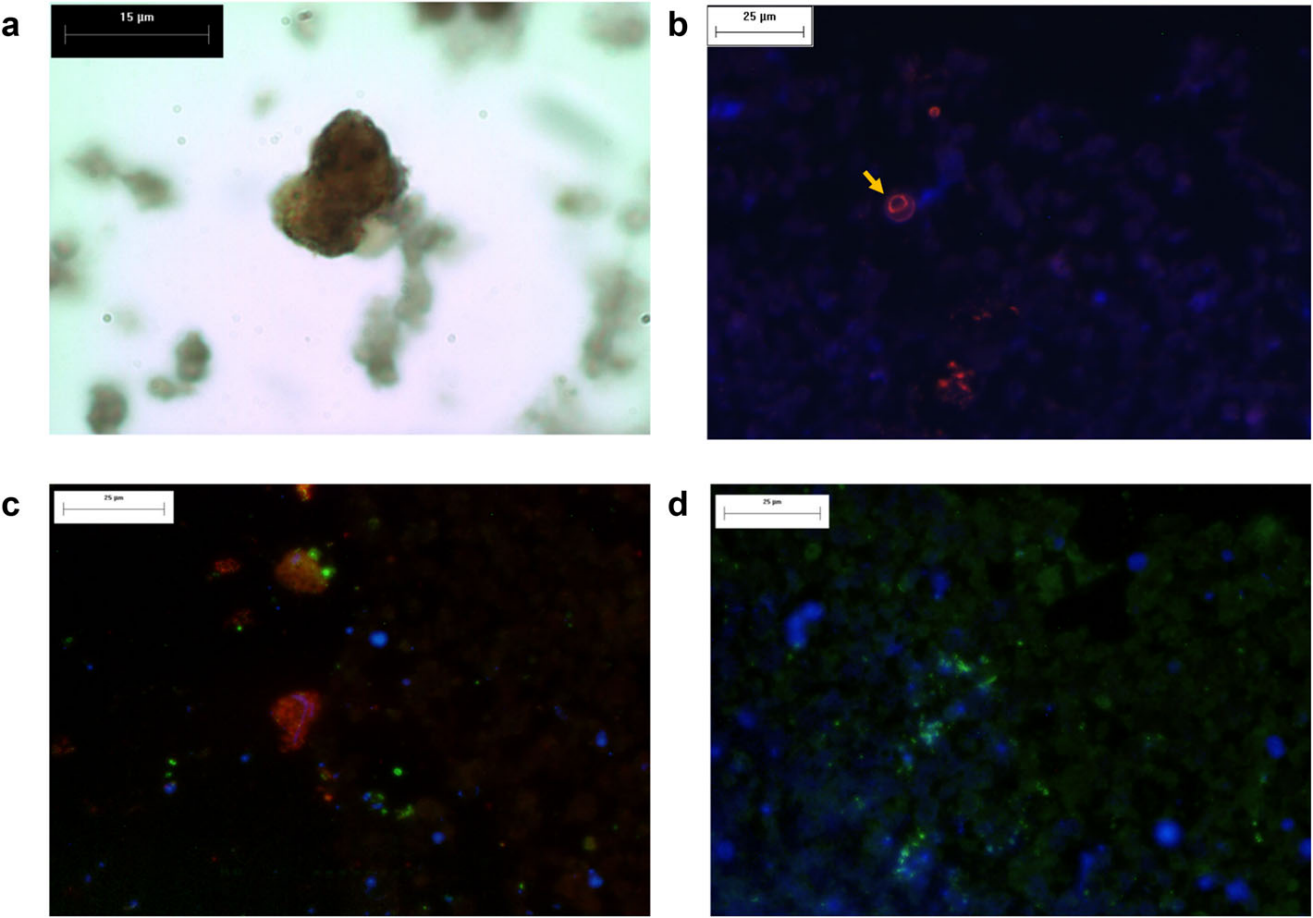
Immunodetection of *E. histolytica* trophozoites in brain tissue by immunohistochemistry and immunofluorescence. Identification of the *E. histolytica* 140 kDa fibronectin (FN)-binding protein (EhFNR) and glial fibrillary acidic protein (GFAP) in brain tissue by immunofluorescence. **a** Amoebic trophozoites stained using peroxidase-labeled rabbit anti-*E. histolytica* polyclonal antibody (X 1000). **b** *E. histolytica* actin cytoskeleton dynamics and distribution in amebic brain abscesses. Actin was stained with rhodamine-phalloidin (1:40, red), forming plate adhesions, as shown by the yellow arrow (X 400). **c** *E. histolytica* trophozoites stained positive for EhFNR (red), GFAP (green) and nuclei (Hoechst 1:1000, blue) in amebic brain abscess tissue (X400). **d** GFAP-immunoreactive cells in brain sections (green) and nuclei (blue) (X400)

Brain biopsy tissue showed a large necrotic area with an amoeboid structure (red arrow) on the periphery of the brain tissue abscess (Fig. 2). The presence of *E. histolytica* trophozoites in cerebral biopsy specimens was confirmed by immunohistochemistry using a rabbit polyclonal anti-*E. histolytica* antibody [11] (Fig. 3a) and mouse anti-140 kDa fibronectin (FN)-binding protein (EhFNR) [12] (Fig. 3c). Furthermore, staining with rhodamine phalloidin revealed amoebic structures rich in actin filaments that formed adhesion plaques and macropinosomes (Fig. 3b, yellow arrows). The rest of the brain tissue was positive for glial fibrillary acidic protein (GFAP) (Fig. 3d) by immunofluorescence.

**Fig. 4:**
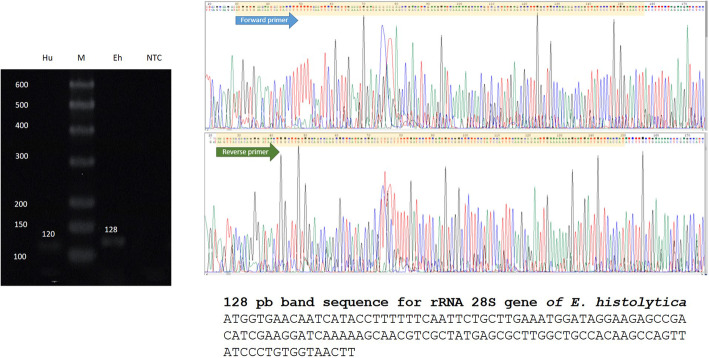
PCR, cloning and sequencing. Total DNA was extracted from 100 mg paraffin-embedded cerebral tissue using the Wizard Genomic DNA purification kit (Promega, Madison, WI, USA). DNA was quantified in a NanoDrop 2000 (Thermo Scientific, Waltham, MA, USA), obtaining an *E. histolytica* 128 bp amplicon for the rRNA gene, which was cloned with the CloneJET PCR Cloning Kit (Thermo Scientific) using a pJET1.2/blunt cloning vector. Then, the ligation mixture was used for transformation of *Escherichia coli* DH5a calcium-competent cells. Plasmid DNA was extracted from heat-shocked cells with the Zyppy Plasmid Miniprep (Zymo Research, Irvine, CA, USA). Clones were analyzed by PCR to verify the insertion of the amplicon into the pJET1.2/blunt vector. The plasmid sequence shows forward and reverse primers (electropherograms) that correspond to the *E. histolytica* rRNA gene sequence. Hu = 120 bp amplicon for human β-actin; M = bp marker; Eh = 128 bp amplicon for the *E. histolytica* rRNA 18 s gene, NTC = no template control

**Fig. 5:**
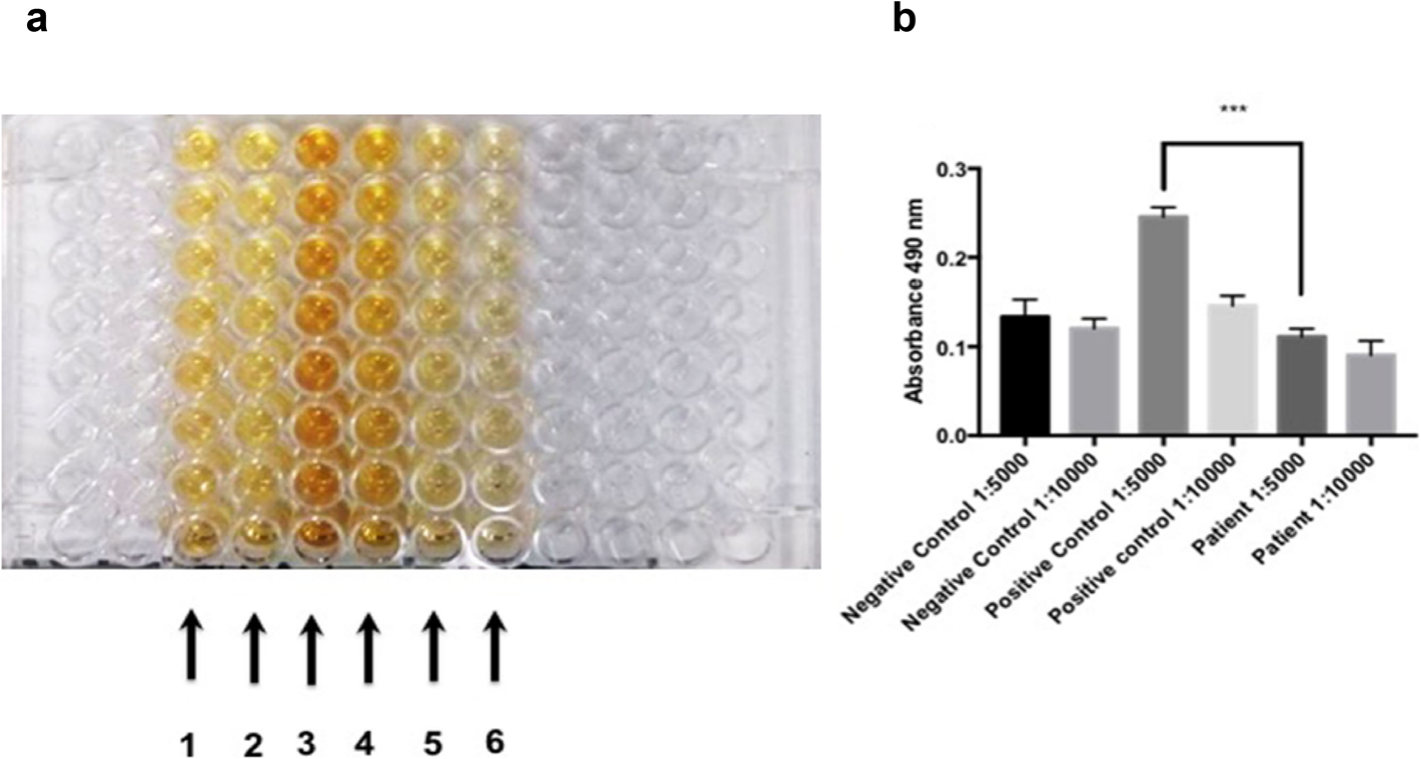
ELISA. **a** The ELISA plate displays a slight reaction to a negative control (1, 1: 5000; 2, 1: 10000). The positive reaction is evident from the application of anti-*E. histolytica* antibodies to the serum of an individual with amoebic liver abscess (3, 1: 5000; 4, 1: 10000). There was a negative reaction to anti-*E. histolytica* antibodies in the serum of the patient under study despite the presence of brain abscesses (5, 1: 5000; 6, 1: 10000). **b** The graph shows the significant difference between the positive control (1:5000) and the patient in the current case study (1:5000) (***) ANOVA

The presence of *E. histolytica* in the cerebral tissue was corroborated by PCR, and an 128 bp amplicon of the *E. histolytica* rRNA gene (NCBI Accession number X65163.1) was cloned from cerebral tissue with the CloneJET PCR Cloning Kit (Thermo Scientific). DNA sequencing was performed in the Unit of Molecular Biology of the Institute of Cellular Physiology (National Autonomous University of Mexico) (Fig. 4). Interestingly, the ELISA of the patient serum did not find IgG antibodies against *E. histolytica* or amoebic proteins. Absorbance data analysis showed a cutoff for the negative control of 186.38; the median for amoebic cerebral abscess patients was 111.5, a number below that of the negative control; however, the median for the positive control was 477.3 (Fig. 5a and b). Based on a diagnosis of amoebic brain abscess, the patient was treated with ceftriaxone (2 g IV every 12 h), metronidazole (750 mg IV every 8 h), and dexamethasone (8 mg IV every 8 h) for 4 weeks, and no antiepileptic drugs were administered. A deteriorating condition led to her readmission to the hospital on October 14, 2018, and she died four days later.

## Discussion and conclusion

Amoebic brain abscess is considered to occur in individuals with associated infections [13]. The current case began with signs of neurological alterations, muscle weakness, loss of memory and disorientation but without fever, diarrhea or amebic damage to the intestines or liver. Due to the absence of associated infections, this case was very different from the cases described by Orbison et al. [4] and Petri and Haque [6].

The surprising inability of the humoral immune response to detect *E. histolytica* prevented the organism from eliminating the trophozoites present in the brain; however, the identification of amoebic trophozoites was performed by applying a specific polyclonal monospecific 140 kDa amoebic protein antibody that acts as a fibronectin receptor [12].

In response to a diagnosis of probable neurocysticercosis and possible hydatid cysts, the patient received metronidazole and dexamethasone during the last 4 weeks of her life. The administration of metronidazole was successful in treating individuals with amoebic cerebral abscesses [7, 8, 14, 15]. However, some cases have been treated with intravenous and oral metronidazole without positive results [9] in patients where treatment is not effective, and the aggravating factor may be the poor state of health of the patient.

However, the use of prednisolone in our patient apparently had a negative effect, consistent with a recent review by Shirley and Moonah [16]. Of 525 case reports of fulminant amoebic colitis, 24 of the subjects received corticosteroid therapy. However, 14 (58%) were incorrectly diagnosed with inflammatory colitis and underwent a very rapid progression of amoebiasis.

In our case, the patient’s clinical features were insidious, and several laboratory and cabinet studies had to be carried out to obtain a precise diagnosis of cerebral amebiasis in such a way that the intravenous metronidazole did not eliminate the parasite and the patient died. Similar cases have been reported by Akhaddar [17]. Furthermore, Bansal et al. [18] and Ehrenkaufer et al. [19] reported a partial resistance of the parasite to the treatment, and Petri and Haque [6] observed that 40–60% of the treated patients maintained the parasite in the colon lumen.

Our patient did not have a history of hepatic or intestinal amoebiasis, and serum analysis was negative for IgG antibodies against *E. histolytica*. This result illustrates the importance of adopting a series of basic laboratory tests, including immunofluorescence, NMRI and PCR, for patients with brain abscesses. Moreover, it must be taken into account that the administration of corticosteroids to such patients has often led to a rapid decline in their condition.

## Data Availability

The most relevant data generated or analyzed during the current study are included in this report. Additional data examined by neurosurgery to eliminate amoebic abscesses are available in the video “Cerebral amebiasis” at https://www.synapse.org/#!Synapse:syn22236751, DOI: 10.7303/syn22236751.1, 10.7303/syn22236751.1

## Abbreviations

*E. histolytica*: *Entamoeba histolytica*
ELISA: enzyme-linked immunosorbent assay
NMRI: nuclear magnetic resonance imaging
CD59: protectin
PCR: polymerase chain reaction
